# Comparison of ^11^C-4′-thiothymidine, ^11^C-methionine, and ^18^F-FDG PET/CT for the detection of active lesions of multiple myeloma

**DOI:** 10.1007/s12149-014-0931-9

**Published:** 2014-11-25

**Authors:** Momoko Okasaki, Kazuo Kubota, Ryogo Minamimoto, Yoko Miyata, Miyako Morooka, Kimiteru Ito, Kiichi Ishiwata, Jun Toyohara, Tomio Inoue, Risen Hirai, Shotaro Hagiwara, Akiyoshi Miwa

**Affiliations:** 1 Division of Nuclear Medicine, National Center for Global Health and Medicine, 1-21-1, Toyama, Shinjuku-ku, Tokyo, 162-8655 Japan; 2 Department of Radiology, National Center of Neurology and Psychiatry, 4-1-1, Ogawahigashi-cho, Kodaira-shi, Tokyo, 187-8551 Japan; 3 Research Team for Neuroimaging, Tokyo Metropolitan Institute of Gerontology, 35-2, Sakae-cho, Itabashi-ku, Tokyo 173-0015 Japan; 4 Departments of Radiology, Yokohama City University Graduate School of Medicine, 3-9, Fukuura, Kanazawa-ku, Yokohama-shi, Kanagawa, 236-0004 Japan; 5 Division of Hematology, National Center for Global Health and Medicine, 1-21-1, Toyama, Shinjuku-ku, Tokyo, 162-8655 Japan

**Keywords:** FDG, Methionine, ^11^C-4′-thiothymidine, PET/CT, Multiple myeloma

## Abstract

**Purpose:**

The aims of this study were to evaluate the possibility of using ^11^C-methionine (^11^C-MET) and ^11^C-4′-thiothymidine (^11^C-4DST) whole-body PET/CT for the imaging of amino acid metabolism and DNA synthesis, respectively, when searching for bone marrow involvement in patients with multiple myeloma (MM) and to compare these findings with those for ^18^F-FDG PET/CT and aspiration cytology.

**Methods:**

A total of 64 patients with MM, solitary plasmacytoma, monoclonal gammopathy of undetermined significance, or an unspecified diagnosis were prospectively enrolled. All the patients underwent three whole-body PET/CT examinations within a period of 1 week. First, the tracer accumulation was visually evaluated as positive, equivocal, or negative for 55 focal lytic lesions visualized using CT in 24 patients. Second, the percentages of marrow plasma cells as calculated using a bone marrow aspiration smear and tracer accumulation were evaluated in the posterior iliac crests of 36 patients.

**Results:**

Among the 55 lytic lesions, the ^11^C-MET and ^11^C-4DST findings tended to reveal more positive findings than the ^18^F-FDG findings. Based on the standard criteria for the diagnosis of active myeloma using the percentage of marrow plasma cells, significant differences were found between the ^18^F-FDG and ^11^C-MET findings and between the ^18^F-FDG and ^11^C-4DST findings, but no significant difference was observed between the ^11^C-MET and ^11^C-4DST findings.

**Conclusion:**

The addition of ^11^C-MET and ^11^C-4DST to ^18^F-FDG when performing PET/CT enabled clearer evaluations of equivocal lesions. Based on cytological diagnostic criteria, ^11^C-MET and ^11^C-4DST were more sensitive than ^18^F-FDG for the detection of active lesions. ^11^C-MET and ^11^C-4DST were more useful than ^18^F-FDG for the detection of active lesions, especially during the early stage of disease.

## Introduction

Multiple myeloma (MM) is characterized by the neoplastic proliferation of plasma cells [1], and the majority of myeloma cells produce monoclonal immunoglobulins or immunoglobulin-related proteins and various proteins causing complications. MM may present with both osseous and extraosseous manifestations and accounts for 1 % of all malignant diseases [2] and about 10 % of hematologic malignancies [3]. Symptoms develop as a result of anemia, immunosuppression, renal failure, hypercalcemia, and bone destruction resulting in frequent pathologic fractures [4]. MM typically evolves from a premalignant condition called monoclonal gammopathy of undetermined significance (MGUS), with M (monoclonal)-protein detected in blood or urine. Typically, end-stage organ damage does not occur in MGUS or in a more progressed condition called smoldering MM. MGUS and smoldering MM are usually not treated [5, 6]. The new Durie/Salmon PLUS staging system has integrated new imaging techniques into a new generation of anatomic and functional myeloma staging [7].

Morphologic imaging techniques, such as X-ray, computed tomography (CT), and magnetic resonance imaging (MRI), show the extent of tumors but not their activity or their viability; thus, these techniques have limitations when assessing the treatment response or early progression.

^18^F-FDG PET/CT is a non-invasive, whole-body imaging method that has been widely used to detect malignant tissues and to monitor treatment response in patients with solid tumors and lymphoma [8, 9]. In addition, the technique is useful for examining the function of the red marrow and for detecting bone marrow involvement in both benign and malignant disorders. Consequently, ^18^F-FDG PET/CT is well recognized as a powerful diagnostic tool for the initial staging of patients with MM [10]. In addition, ^18^F-FDG PET/CT is also useful for evaluating the response to therapy [11], such as the restaging of MM [12].

However, ^18^F-FDG accumulation in the areas of inflammation or infection may obscure accurate evaluations of the therapeutic effects on tumor tissues [13]; hence, false-negative results have been reported for ^18^F-FDG, especially in patients with early stage MM [11]. The use of new tracers capable of compensating for the limitations of ^18^F-FDG could be very helpful for detecting active myeloma lesions more accurately than with ^18^F-FDG alone.

The use of ^11^C-methionine (^11^C-MET) and ^18^F-fluoro-deoxy-L-thymidine (^18^F-FLT) has been reported for the evaluation of MM. ^18^F-FDG, ^11^C-MET, and ^18^F-FLT have different mechanisms of tracer uptake, enabling the visualization of the metabolic status of glucose, amino acid metabolism, and proliferative activity, respectively, in bone marrow and extramedullary lesions [14].

^11^C-MET is a widely used tracer for the imaging of brain tumors [15]. An increase in ^11^C-MET uptake in the bone marrow reportedly reflects an increase in cellular proliferation and protein synthesis [16]. MM is characterized by the neoplastic proliferation of plasma cells and the production of monoclonal immunoglobulins. Dankerl et al. reported that the increase in methionine uptake in plasma cells is the basis for the imaging of active MM using ^11^C-MET PET/CT, and peripheral bone marrow expansion has been observed in MM patients. Extramedullary MM can also be detected and localized with a high sensitivity using ^11^C-MET PET/CT [16].

^18^F-FLT is a tracer that monitors the activity of thymidine kinase one and its uptake and hence is related to DNA synthesis, which is a surrogate marker for cellular proliferation. Therefore, ^18^F-FLT may help in differentiating old and inactive lytic lesions from foci of rapidly proliferating MM cells, which could be potential targets for local radiation treatment [14].

Alternatively, Toyohara et al. developed [methyl-^11^C] 4′-thiothymidine (^11^C-4DST) as a novel tracer for imaging cell proliferation. ^11^C-4DST is a promising tracer because after it has been incorporated into DNA, the occurrence of labeled nucleotide dephosphorylation (which can be an issue with ^18^F-FLT) is relatively rare [17]. This irreversible nature of ^11^C-4DST is expected to contribute to a more sensitive tumor uptake than ^18^F-FLT. Indeed, the tumor uptake of ^11^C-4DST was higher than that of ^3^H-FLT and was correlated with the DNA synthesis level in animal models [18]. Initial clinical trials of ^11^C-4DST have demonstrated its safety, radiation dosimetry, and application for brain tumor imaging [19].

Furthermore, Minamimoto et al. applied ^11^C-4DST PET/CT to proliferation imaging in non-small cell lung cancer and demonstrated a strong correlation between ^11^C-4DST uptake by tumor tissues before surgery and the MIB-1 index of the surgical pathology findings (a standard marker of proliferation). A linear regression analysis indicated that the SUVmax for ^11^C-4DST was not significantly correlated with the microvessel density, as determined using CD31 staining [20]. Thus, ^11^C-4DST and blood flow are not correlated, whereas ^11^C-4DST is correlated with cell proliferation. Based on these conclusions, we hypothesized that ^11^C-4DST might be capable of detecting the proliferation status of MM more sensitively than ^18^F-FLT. Hence, we selected ^11^C-4DST as a representative marker of in vivo proliferation.

The aim of the present study was to evaluate the potential of whole-body ^11^C-4DST and ^11^C-MET PET/CT imaging for the detection of bone marrow involvement in patients with MM and to compare the results with those obtained using ^18^F-FDG.

## Materials and methods

### Patients

Between October 2010 and October 2011, a total of 64 patients with MM or MGUS (40 men, 24 women; mean age, 58.3 years; range 33–84 years) were prospectively enrolled in this study (Table 1). All the patients were diagnosed as having MM or MGUS based on the criteria defined by the International Myeloma Working Group. Twenty-one patients were previously untreated, and 43 patients were restaged after treatment. All the patients underwent three whole-body PET-CT scans with ^18^F-FDG, ^11^C-MET, and ^11^C-4DST within a period of 1 week.

**Table 1:** Characteristics of total patients, aspiration patients, and CT patients

Parameter	Patients		
	Total^a^	CT	Aspiration
Subjects (*n*)	64	24	36
Before therapy (*n*)	21	6	12
Restaging (*n*)	43	18	24
Age (years, mean ± SD)	58.3 ± 10.7	56.3 ± 11.2	59.6 ± 9.47
Range	33–84	36–80	36–80
Sex (male/female)	40/24	16/8	21/15
Type of myeloma			
Heavy chain component			
IgG	36	15	20
IgA	8	3	5
IgD	1	0	0
IgM	1	0	0
BJP	9	3	6
Non-secretory	1	0	1
MGUS	3	0	1
Solitary plasmacytoma	4	3	1
Light chain component			
Kappa	33	15	19
Lambda	19	5	11
Unspecified	3	1	1
Initial stage (Durie/Salmon)			
I	12	2	6
II	14	5	8
III	28	13	17
A	45	17	27
B	7	3	5
Unspecified	2	1	1
MGUS	3	0	3
Solitary plasmacytoma	5	1	1
Non-secretory	1	0	1
Unspecified	1	0	0

*BJP* Bence Jones protein, *MGUS* monoclonal gammopathy of undetermined significance

^a^Total = CT + aspiration + other

MM is not a solid tumor but a hematological malignancy that appears as a mottled lesion distributed in the bone marrow. Consequently, biopsies of MM lesions are difficult to perform, and the tumor range can be difficult to discriminate. Thus, methods for evaluating MM require special consideration.

To compare lesion visualization, we focused on focal lytic lesions visible using CT for which the lesion localization was obvious and the tracer uptake by the lesions in the three PET studies could be easily and accurately compared. Twenty-four patients who had focal lytic lesions visible using CT were enrolled in Study 1. The remaining patients did not have focal lytic lesions, but instead had diffuse lesions or normal findings when examined using CT.

**Fig. 1:**
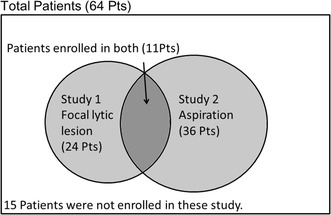
Sixty-four patients underwent three whole-body PET-CT scans using 18F-FDG, 11C-MET, and 11C-4DST within a period of 1 week. Twenty-four patients with focal lytic lesions visible on CT images were enrolled in Study 1. The remaining patients did not have focal lytic lesions. Thirty-six patients underwent bone marrow aspiration cytology within 1 week of the three PET/CT scans and were enrolled in Study 2. Eleven patients were enrolled in both studies

To verify tracer uptake by the lesions, we focused on lesions for which bone marrow aspiration cytology was performed. Thirty-six patients underwent bone marrow aspiration cytology within 1 week of the three PET/CT scans (Study 2). The remaining patients did not undergo bone marrow aspiration cytology within 1 week of the PET/CT scans. Fifteen patients were not enrolled in either study (Fig. 1).

The present study was approved by the institutional review board, and written informed consent was obtained from all the subjects.

### PET/CT examination

An in-house cyclotron and automated synthesis system (F200 Sumitomo Heavy Industries, Tokyo, Japan) produced the ^18^F-FDG. ^11^C-MET and ^11^C-4DST were synthesized as reported previously [19, 21]. The PET/CT images were obtained using two PET/CT systems (29 patients: Biograph 16; Siemens, München, Germany; and 35 patients: Discovery PET/CT 600; GE Healthcare, Fairfield, CF), with measurements obtained from the vertex to the toe 60 min after the intravenous injection of ^18^F-FDG (5 MBq/kg), 20 min after the injection of ^11^C-MET (370 MBq), or 40 min after the injection of ^11^C-4DST (370 MBq). The SUV consistency for these two PET/CT scanners was validated using a phantom study. Low-dose CT was performed first for attenuation correction and image fusion. Emission images were acquired in a three-dimensional mode for 2 min per bed position using both PET/CT scanners. The PET/CT data were reconstructed using a Gaussian filter with an ordered subset expectation maximization algorithm (3 iterations, 8 subsets for Biograph 16, 3 and 16 subsets for Discovery PET/CT 600, according to the manufacturers’ recommendations).

The radiation exposure from ^11^C-labeled radiopharmaceuticals is much lower than that from ^18^F-labeled radiopharmaceuticals. The estimated effective dose for ^11^C-4DST is 1.6 mSv [19], that for ^11^C-MET is 2.1 mSv, that for ^18^F-FDG is 7 mSv [22], and that for low-dose CT is 1.4–3.5 mSv. Therefore, the total effective dose delivered during all the PET/CT examinations was about 14.9–21.2 mSv. We felt that the radiation exposure from the PET/CT examinations performed in the present study was acceptable when their impact on the therapeutic strategy was considered.

### PET data analysis

Xeleris (GE Healthcare) and e-Soft (Siemens) workstations were used for image analysis. The physiological uptake of ^11^C-MET is seen in the gastrointestinal tract, liver, pancreas, urinary tract, and salivary glands, as reported by Nishizawa et al. [15]. A high physiological ^11^C-4DST uptake is observed in the salivary glands, liver, spleen, kidneys, bladder, and bone marrow. In contrast, the brain, lungs, myocardium, muscle, and blood pool exhibit a low physiological ^11^C-4DST uptake [20]. The SUVmax of ^11^C-4DST in the normal bone marrow is higher than that of ^18^F-FDG (lumbar vertebrae 2–4, ilium, proximal humeri, and proximal femurs), and the SUVmax of ^11^C-MET falls between these two values. Based on this normal background, the active accumulations of ^18^F-FDG, ^11^C-MET, and ^11^C-4DST in the bone marrow lesions were evaluated.

Two experienced nuclear medicine physicians visually evaluated all the PET/CT scans for tracer accumulation in the lesions (positive, equivocal, or negative); the maximum standardized uptake value (SUVmax) was also recorded for each lesion. If the results of the two physicians differed, the physicians discussed the findings and reached a consensus. To evaluate the bone marrow lesions on the ^11^C-MET and ^11^C-4DST PET/CT images, the scale and window of the monitor display for these PET/CT images had to be adjusted so that the pathological uptake could be visualized with a better contrast against the high physiological uptake in the normal bone marrow. Before the start of this study, the physicians underwent training that included images from more than ten patients who had non-MM without bone diseases, since Nakamoto et al. [23] reported that suspicious lesions, including those in the bone marrow, could be clearly depicted using a proper display window and level in their ^11^C-MET PET/CT study. Focal accumulation that was higher than the background was regarded as being positive, no accumulation compared with the background was regarded as being negative, and accumulation with the same level as the background was regarded as being equivocal.

### Focal lytic lesions

In 24 patients (before receiving therapy, 6 patients; after receiving therapy, 18 patients), a total of 55 focal lytic lesions (before receiving therapy, 10 lesions, after receiving therapy, 45 lesions) were detected using CT when PET/CT was performed, but no diffuse lesions were detected. The sizes of the 55 focal lytic lesions were 23.7 ± 13.8 mm (range 6–70 mm), which was sufficiently large to be evaluated using low-dose CT and PET/CT.

### Comparison to marrow plasma cells cytology

The percentages of marrow plasma cells in the posterior iliac crests were calculated using bone marrow aspiration smears in 36 patients (Table 2) within 1 week before or after the three PET/CT studies. Eleven of the patients were also included in the first study. According to the criteria of the International Myeloma Working Group, a bone marrow clonal cell percentage of more than 10 % is regarded as a positive pathology for active myeloma and should be regarded as the gold standard for diagnosis [24].

**Table 2:** Correlation between cytology and PET/CT findings

Pt No	Plasma cell (%)	FDG	MET	4DST
1	84	○	○	○
2	81.5	○	○	○
3	71.2	○	○	○
4	67.4	○	○	○
5	58.8	○	○	○
6	45.4	×	○	○
7	43.6	△	○	○
8	36	△	○	○
9	30.4	×	○	○
10	21.4	×	×	×
11	21	×	×	○
12	17	×	○	○
13	12	△	△	○
14	10.6	×	○	○
15	10	△	△	○
16	8.8	×	×	×
17	8.5	×	×	×
18	8.2	×	×	×
19	7.8	×	×	×
20	4.4	△	○	○
21	2.6	×	×	×
22	1.6	×	×	×
23	1.2	○	○	○
24	1	×	×	×
25	1	×	×	×
26	0.8	×	×	×
27	0.6	×	×	×
28	0.6	×	×	△
29	0.4	×	×	×
30	0.4	△	△	△
31	0.4	×	×	×
32	0.4	×	×	×
33	0.3	×	×	×
34	0.2	×	×	×
35	0	△	○	○
36	0	△	△	△

Note 10 % of the plasma cells in the cytology specimen is the threshold for active myeloma

PET/CT findings: positive ○, equivocal △, negative ×

We evaluated the accumulation of ^18^F-FDG, ^11^C-MET, and ^11^C-4DST in the posterior iliac crests from where the bone marrow samples were obtained. The tracer accumulation was evaluated visually as positive, equivocal, or negative uptake. Even if abnormal accumulation was visible in lesions other than the posterior iliac crests, a positive-uptake evaluation was not made. Also, when an artifact from the bone marrow puncture was observed, the artifact was carefully excluded from the evaluation. Then, we compared the sensitivity, specificity, positive predictive value, negative predictive value, and accuracy rate of PET/CT using each of the three tracers.

### Statistical analysis

The significance of the differences in the accumulation of the three tracers was determined using the area under the curve with a receiver operating characteristic (ROC) analysis. The significance of the differences between bone marrow aspiration and the accumulation of each of the three tracers was determined using the Fisher exact test. *P* values less than 0.05 were considered to be statistically significant.

## Results

### Focal lytic lesions

Both before and after therapy, the number of equivocal lesions observed using ^18^F-FDG was larger than that observed using ^11^C-MET or ^11^C-4DST. For ^11^C-4DST, ^11^C-MET, and ^18^F-FDG, the highest SUVmax values were observed, in order, for positive, equivocal and negative lesions (Table 3). Among the patients who were examined after therapy, in particular, ^11^C-MET or ^11^C-4DST was capable of detecting positive lesions more frequently than ^18^F-FDG.

**Table 3:** Accumulation of ^18^F-FDG, ^11^C-MET, and ^11^C-4DST in lytic lesions on CT

	Number of lesions				SUVmax		
	Positive	Equivocal	Negative	Total	Positive	Equivocal	Negative
Patients examined before therapy							
^18^F-FDG	6	2	2	10	4.00 ± 1.63	1.98	1.55
^11^C-MET	10	0	0	10	5.74 ± 2.15	–	–
^11^C-4DST	8	0	2	10	15.8 ± 9.79	–	3.5
Patients examined after therapy							
^18^F-FDG	27	8	10	45	3.20 ± 1.70	2.05 ± 0.28	1.55 ± 0.41
^11^C-MET	39	3	13	45	5.01 ± 2.40	3.89 ± 1.87	2.19 ± 1.28
^11^C-4DST	32	1	12	45	6.44 ± 3.07	4.25	3.39 ± 3.29
Total patients							
^18^F-FDG	33	10	12	55	3.35 ± 1.70	2.03 ± 0.26	1.54 ± 0.38
^11^C-MET	39	3	13	55	5.19 ± 2.40	3.89 ± 1.87	2.19 ± 1.28
^11^C-4DST	40	1	14	55	8.30 ± 6.24	4.25	3.40 ± 3.02

Data are the mean ± SD

*SUVmax* maximum standardized uptake values

**Fig. 2:**
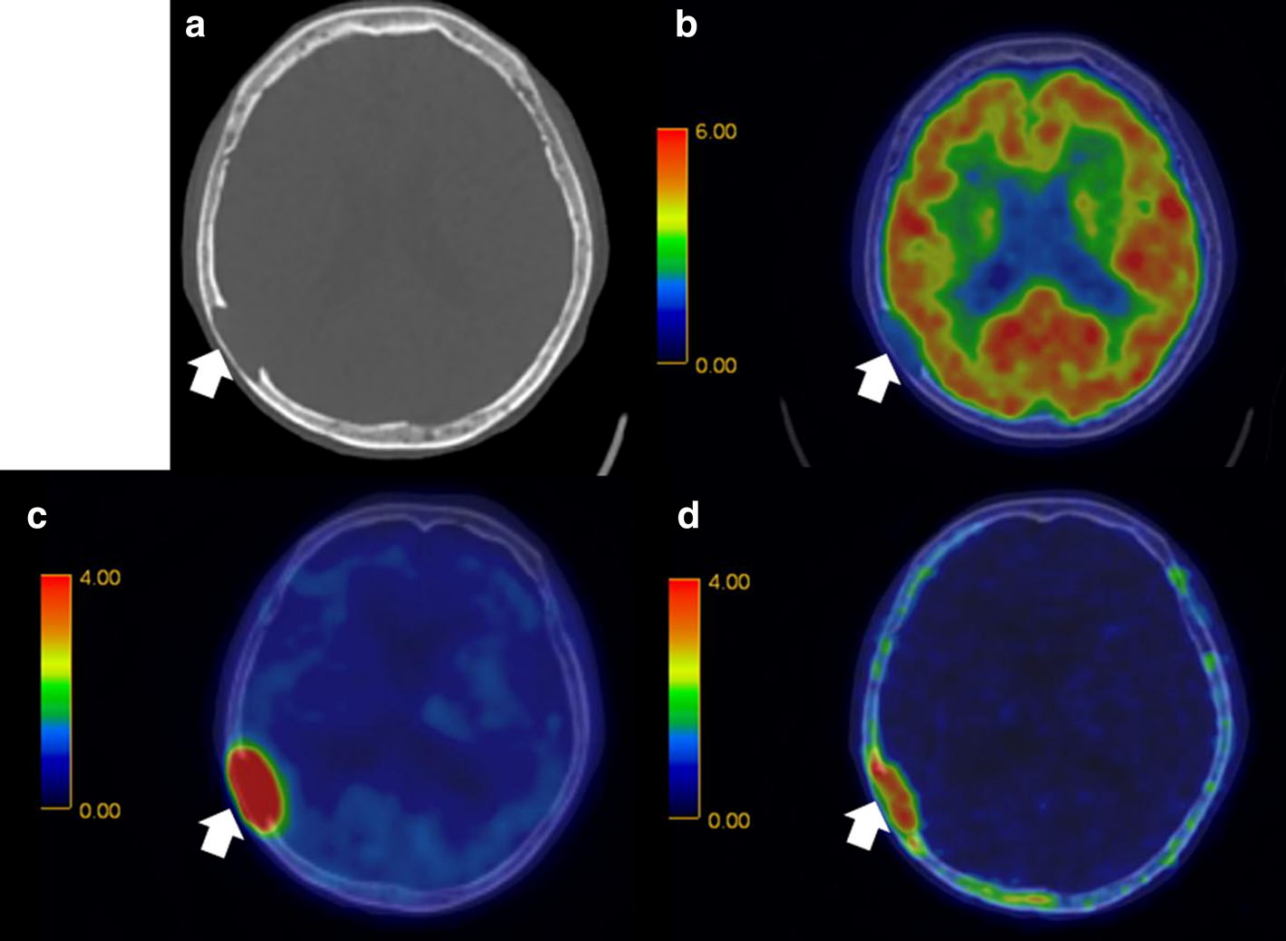
Fusion images of **a** CT, **b** ^18^F-FDG, **c** ^11^C-MET, and **d** ^11^C-4DST obtained in a 79-year-old woman with multiple myeloma (IgG-κ). The *arrow* shows an osteolytic lesion. ^11^C-MET PET/CT and ^11^C-4DST PET/CT detected activity in the skull lesion, whereas ^18^F-FDG PET/CT could not detect any activity because of normal brain accumulation

**Fig. 3:**
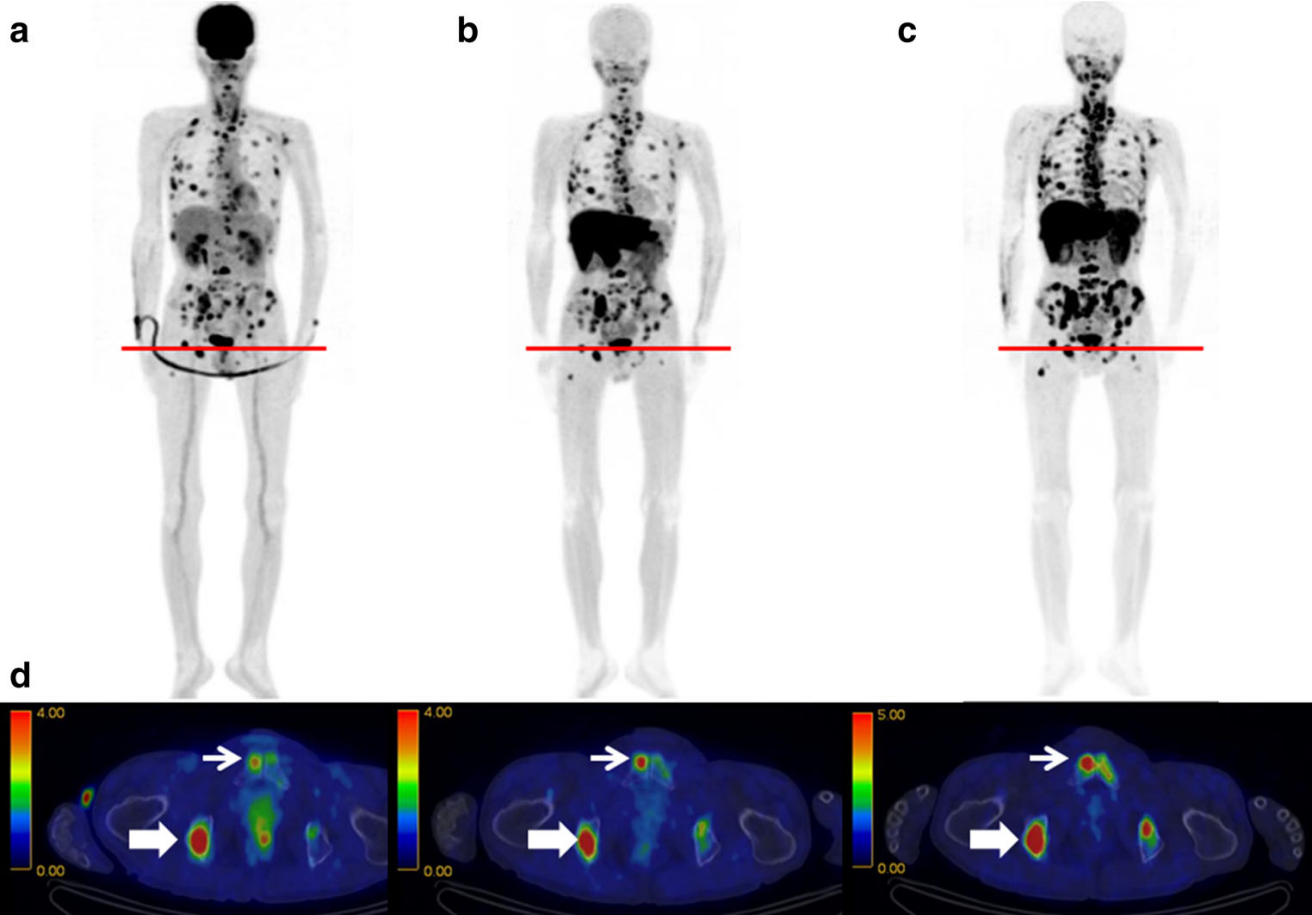
Maximum intensity projection images and fusion images. **a** ^18^F-FDG, **b** ^11^C-MET, and **c** ^11^C-4DST PET images obtained in a 63-year-old man (Patient 1) with MM (IgA-κ). Numerous active lesions are visible in the three maximum intensity projection images. The fusion images are for the cross-section at the level of the *red lines* (**d**). The lesion in the right ischium (*bold arrow*) was positive on all three PET scans. However, the lesion in the right pubis (*narrow arrow*) was only positive on the ^11^C-MET PET and ^11^C-4DST PET scans and was equivocal on the ^18^F-FDG PET scan (color figure online)

**Fig. 4:**
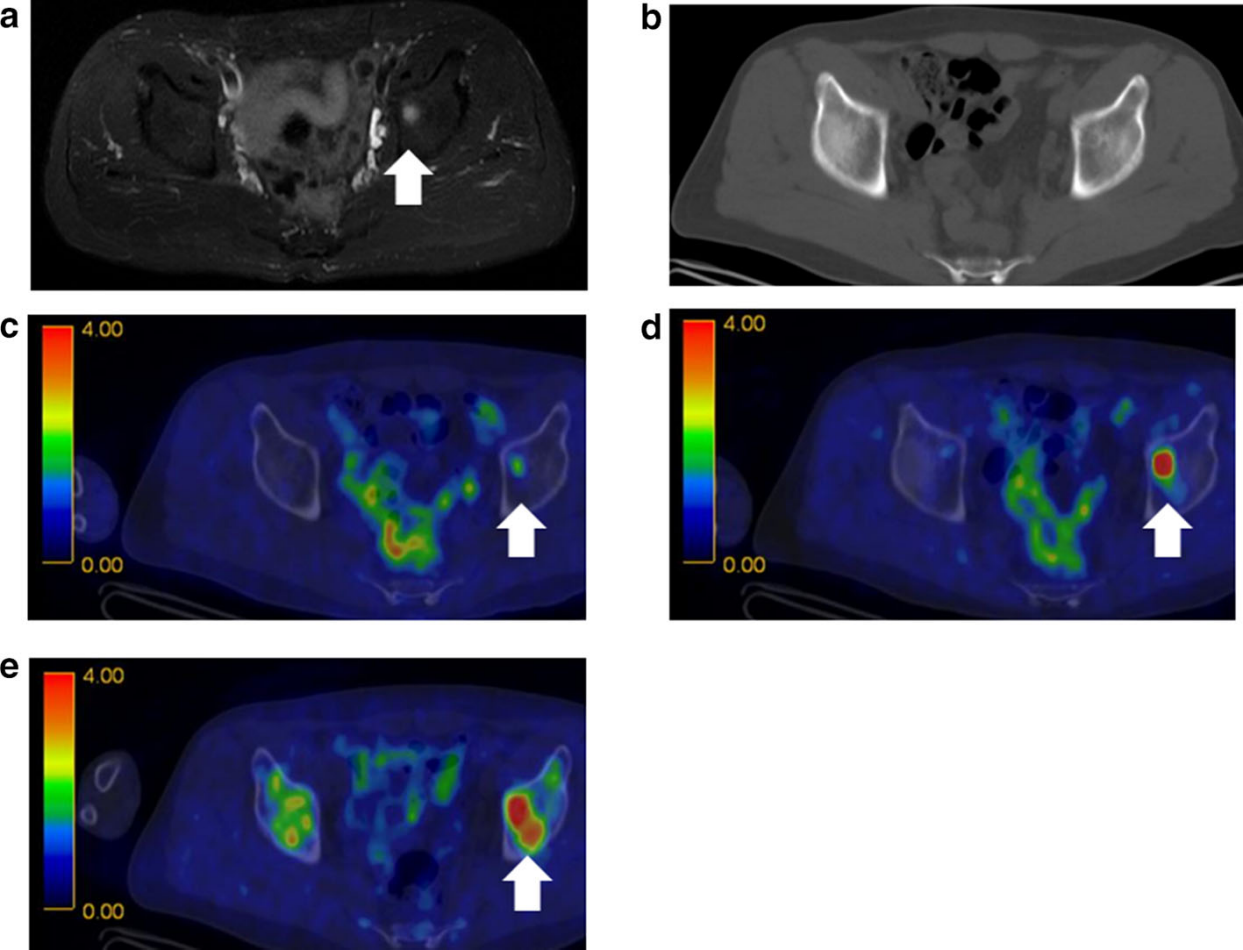
**a** CT and **b** MRI fusion images of **c** ^18^F-FDG PET/CT, **d** ^11^C-MET PET/CT, and **e** ^11^C-4DST PET/CT obtained in a 63-year-old man. The acetabular lesion was positive on the MRI, ^11^C-MET PET/CT, and ^11^C-4DST PET/CT images, equivocal on the ^18^F-FDG PET scans, and negative on the CT images

^18^F-FDG was rarely able to detect skull lesions because of the high physiological accumulation in the brain, whereas ^11^C-MET and ^11^C-4DST were capable of clearly detecting skull lesions because of their low accumulation in the brain (Fig. 2). A typical MM patient with multiple active lesions is sh own in Fig. 3. ^11^C-MET and ^11^C-4DST detected positive lesions, whereas ^18^F-FDG detected an equivocal lesion. The lesion was positive when evaluated using MRI and negative when evaluated using CT (Fig. 4).

### Comparison to marrow plasma cells cytology

A Fisher exact test demonstrated a significant correlation between positive uptake on the PET/CT scans and a positive pathology of the bone marrow plasma cells. The *P* values of ^11^C-MET and ^11^C-4DST were lower than that of ^18^F-FDG. The diagnostic potentials of the three tracers are as described below. ^11^C-4DST showed the highest sensitivity. The specificity of the three tracers was comparable (Table 4). The ROC analysis showed statistically significant differences between ^18^F-FDG and ^11^C-MET and between ^18^F-FDG and ^11^C-4DST (Table 5). The area under the ROC curves for ^11^C-MET and ^11^C-4DST were greater than that for ^18^F-FDG. The other three indices for ^11^C-MET and ^11^C-4DST were larger than those for ^18^F-FDG. But no statistically significant differences in the positive predictive value, negative predictive value, and accuracy rate were observed among the three tracers when examined using a Chi square test (*P* > 0.05).

**Table 4:** Contingency correlating PET/CT positivity with disease activity according to bone marrow aspiration and diagnostic results of PET/CT using each of the three tracers

PET/CT finding	Bone marrow aspiration		*P* value
Fisher exact test	Positive	Negative	
^18^F-FDG			
Positive	9	5	0.03
Negative	6	16	
^11^C-MET			
Positive	13	5	0.0005
Negative	2	16	
^11^C-4DST			
Positive	14	6	0.0001
Negative	1	15	

	^18^F-FDG	^11^C-MET	^11^C-4DST
Sensitivity	60.0	86.7	93.3
Specificity	76.1	76.1	71.4
Positive predictive value	64.3	72.2	70.0
Negative predictive value	72.7	88.9	93.7
Accuracy rate	69.4	80.6	80.6

A Fisher exact test demonstrated a significant correlation between positive uptake on the PET/CT scans and a positive pathology of the bone marrow plasma cells

**Table 5:** Area under the receiver operating characteristic curve

Area	Difference	
^18^F-FDG		
0.681 (95 % CI, 0.522–0.840)		
^11^C-MET		
0.814 (95 % CI, 0.685–0.943)	vs. ^18^F-FDG *P* = 0.02	
^11^C-4DST		
0.824 (95 % CI, 0.705–0.942)	vs. ^18^F-FDG *P* = 0.03	vs. ^11^C-MET *P* = 0.81

*CI* confidence interval

In patients with more than 58 % plasma cells, all the PET/CT data showed a positive uptake. However, in patients with 10–30 % plasma cells, ^11^C-MET and ^11^C-4DST detected larger numbers of positive-uptake lesions than ^18^F-FDG (Table 2).

All three PET/CT scans were negative for all three MGUS patients. Extramedullary lesions were not evaluated in the present study.

## Discussion

We demonstrated the usefulness of ^11^C-4DST and ^11^C-MET PET/CT imaging, compared with ^18^F-FDG PET/CT imaging, in patients with MM. ^11^C-4DST and ^11^C-MET provided clearer findings than ^18^F-FDG for lytic lesions visible using CT. Furthermore, ^11^C-4DST and ^11^C-MET had higher diagnostic accuracies than ^18^F-FDG, when compared using iliac crest biopsy data.

In the first study, ^11^C-4DST and ^11^C-MET provided clearer findings than ^18^F-FDG when evaluating whether lytic lesions detected using CT were active or inactive. ^11^C-4DST and ^11^C-MET showed fewer equivocal accumulations than ^18^F-FDG. Osteolytic lesions are more commonly found in the axial skeleton, skull, shoulder girdle, proximal humeri, ribs, and proximal femurs [25]. ^11^C-4DST and ^11^C-MET were useful for evaluating the tumor activities of these lesions. As shown in Fig. 2, the disease activities of skull lesions can typically be successfully evaluated using ^11^C-4DST and ^11^C-MET, but not ^18^F-FDG. ^11^C-4DST and ^11^C-MET accumulate in myeloma lesions of the peripheral long bones. Dankerl et al. [16] reported that peripheral bone marrow expansion could be observed using ^11^C-MET PET/CT. In accordance with these findings, ^11^C-4DST and ^11^C-MET may be more useful for evaluating MM lesions in peripheral long bones than ^18^F-FDG. Furthermore, Nakamoto et al. reported that ^11^C-MET provided clearer positive findings in some patients, compared with ^18^F-FDG. Therefore, ^11^C-MET was considered to be useful for determining the therapeutic strategy, especially when the ^18^F-FDG findings were equivocal or indeterminate [23]. Thus, ^11^C-4DST and ^11^C-MET seem to be more useful for assessing the disease activities of lytic lesions detected using CT, compared with ^18^F-FDG. Furthermore, all the negative lesions were observed as such using ^11^C-4DST, ^11^C-MET, and ^18^F-FDG.

In the second study, we demonstrated that ^11^C-4DST and ^11^C-MET had higher diagnostic accuracies than ^18^F-FDG. An iliac crest biopsy is the standard method for determining bone marrow infiltration by plasma cells [26]. In smoldering MM (SMM), bone marrow biopsies reveal a 10–30 % diffuse infiltration of plasma cells, while the infiltration is less than 10 % in MGUS [5, 27] with no evidence of MM. Therefore, we evaluated the iliac crests in patients in whom a pathological diagnosis was obtained using ^18^F-FDG, ^11^C-4DST, and ^11^C-MET. A statistical examination was difficult to perform because the number of patients was relatively small, but ^11^C-MET and ^11^C-4DST seemed to be more sensitive than ^18^F-FDG in patients who had not yet received therapy.

In this study, all three PET/CT scans were negative in all three MGUS patients. In patients with MGUS, marrow plasma cells account for less than 10 %, while in myeloma, the bone marrow clonal cells account for no less than 10 % [24]. Both SMM and MGUS are typically not treated, but the prognoses differ. In MGUS, the overall risk of progression is about 1 % per year [28], and the median duration of MGUS and SMM before a diagnosis of myeloma is 81 and 23 months, respectively [29]. Therefore, it is important to distinguish SMM and MGUS.

In this study, extramedullary lesions were not evaluated. However, the ability of PET/CT to evaluate the whole body in a single procedure and the potential to detect medullary and extramedullary lesions during a single examination are important advantages over standard imaging techniques, such as MRI, CT, or radiographs [11]. Dankerl et al. reported that extramedullary MM was sensitively detected and localized using ^11^C-MET. The evaluation of extramedullary lesions is important because these lesions are often difficult to detect but have a major impact on the prognosis. Nakamoto et al. reported a high level of ^11^C-MET uptake in normal liver and pancreas; however, this situation is unlikely to cause false-negative findings because it is unusual to have extramedullary lesions in these organs [25].

When evaluating MM, diffuse lesions are more difficult to evaluate than focal lesions. The uptake of ^18^F-FDG in the skeleton is caused by the activation of hematopoietic marrow, and its pattern and amount can vary with age and with the levels of marrow function, such as the level of function during recovery after chemotherapy or when subjected to the effect of granulocyte colony stimulating factor at the time of the PET/CT examination [30]. Because ^11^C-4DST can be used to evaluate DNA synthesis [20], it can accumulate in active hematopoietic marrow. Thus, it may be difficult to distinguish diffuse MM lesions from hematopoietic marrow. A means of evaluating diffuse MM lesions should be a topic of future PET/CT studies.

A whole-body survey for active lesions is a unique advantage of PET/CT, and modern image processing techniques can minimize artifacts from metal prostheses. Thus, PET/CT has the potential to become a standard modality for the staging of MM.

^11^C-4DST and ^11^C-MET are better at detecting active lesions than ^18^F-FDG. Therefore, the Durie/Salmon PLUS staging results determined using ^11^C-4DST and ^11^C-MET may differ from those determined using ^18^F-FDG. However, the validation of a new staging method requires prognostic observation over a long observation period. The question of which tracer is the best for evaluating the viability of MM will require further observation. We are planning to evaluate this matter in a separate study.

## Conclusion

^11^C-4DST and ^11^C-MET are useful for detecting bone marrow involvement in patients with MM, especially at an early stage, in a manner that is more clearly and more accurately than that using ^18^F-FDG.
